# Diabetic retinopathy is not associated with carbonic anhydrase gene polymorphisms

**Published:** 2009-06-13

**Authors:** S. Abhary, K.P. Burdon, A. Gupta, N. Petrovsky, J.E. Craig

**Affiliations:** 1 Department of Ophthalmology, Flinders Medical Centre and Flinders University, Adelaide, Australia; 2 Department of Ophthalmology, Royal Adelaide Hospital, Adelaide, Australia; 3 Department of Endocrinology, Flinders Medical Centre and Flinders University, Adelaide, Australia

## Abstract

**Purpose:**

Carbonic anhydrase is elevated in the vitreous of patients with proliferative diabetic retinopathy (PDR). This study aimed to determine if common polymorphisms in the carbonic anhydrase (*CA*) gene influence susceptibility to diabetic retinopathy (DR).

**Methods:**

In this multicentered study, a total of 235 control subjects with no DR, 158 subjects with nonproliferative DR (NPDR), 132 with proliferative DR (PDR), and 93 with clinically significant macular edema (CSME) were recruited. Blinding DR was defined as severe NPDR, PDR or CSME. DR subjects were drawn from both type 1 diabetes mellitus (T1DM) and type 2 diabetes mellitus (T2DM) populations. Ten tag single nucleotide polymorphisms were selected to cover the majority of genetic diversity across the *CA* gene.

**Results:**

After adjustments were made for sex, disease duration, and HbA_1_c, no associations were found between any *CA* polymorphisms or haplotypes with any type of retinopathy in T1DM or T2DM.

**Conclusions:**

Sequence variation in *CA* is not associated with the risk of developing retinopathy in T1DM or T2DM and increases the likelihood that elevated vitreous CA may be a consequence rather than cause of DR. Further genetic studies are required to have a better understanding of the pathogenesis of this debilitating diabetic complication.

## Introduction

Diabetic retinopathy (DR) is a sight-threatening microvascular complication of diabetes mellitus (DM) and a major cause of morbidity in individuals with diabetes. With global incidence of DM predicted to double over the next two decades [1], consequent DR frequency has also been predicted to rise [2,3].

There are several well established risk factors for DR, namely hyperglycemia, diabetes duration, and systemic hypertension [4-10]. There is also increasing evidence supporting a genetic component in DR susceptibility given the heterogeneity of DR in subjects with equally poor glycemic control. Several studies, including the Diabetes Control and Complications Trial, have provided evidence for a familial tendency toward DR development, independent of associated risk factors [11-14].

Vitreous humor from PDR patients has been found to contain up to 50 different proteins [15,16], with several studies reporting higher levels of vascular endothelial growth factor and erythropoietin in PDR vitreous when compared to vitreous of nondiabetic patients [17-19]. Carbonic anhydrase (CA) is a widely expressed enzyme in humans that catalyzes the conversion of carbon dioxide to bicarbonate and protons. It thereby plays an important role in acid-base balance [20] and has also been isolated in the human retina [21,22]. Gao et al. [23] recently undertook a proteomic analysis of vitreous from individuals with diabetes and found the CA concentration in individuals with proliferative diabetic retinopathy (PDR) patients to be significantly higher than in control participants without DM or individuals with DM but without DR. In support of a pathological role for CA in DR, intravitreal injection of CA in rats induced retinal fluorescein leakage and retinal edema that was inhibited by coinjection of acetazolamide (a specific CA inhibitor). Intravitreal CA injection increased retinal vascular permeability through increasing vitreous pH, leading to activation of the kallikrein-kinin system. Thus, the CA pathway is potentially important in the development of DR (and especially macular edema), which is characterized in part by increased vascular permeability and retinal edema.

Given the evidence indicating a potential role of CA in DR pathogenesis, the aim of this study was to determine if common polymorphisms in the *CA* gene might contribute to DR susceptibility, for example by influencing CA expression levels or enzyme activity. To our knowledge, this is the first study to examine this potential association.

## Methods

Patients with DR were recruited from the ophthalmology and endocrine clinics of three tertiary hospitals in metropolitan Adelaide, Australia. Ethics approval was obtained from the Human Research Ethics Committees of each hospital, and written informed consent was received from each participant. Enrolled were 554 individuals with DM: 190 with type 1 (T1DM) and 364 with type 2 diabetes mellitus (T2DM) of Caucasian European descent. All participants were over 18 years of age and were required to have either T1DM or T2DM of at least 5 years duration, necessitating oral hypoglycemic or insulin treatment.

Retinopathy status was graded according to the Early Treatment and Diabetic Retinopathy Study criteria [24], and retinopathy status for the worst eye was used in the analyses. Blinding DR was classified as severe nonproliferative DR (NPDR), PDR or clinically significant macular edema (CSME). A detailed questionnaire was conducted, obtaining information regarding ethnicity, diabetes related risk factors and systemic and ocular complications. Blood pressure and body mass index (BMI) were measured. Renal function tests (serum creatinine, urine albumin, and albumin:creatinine ratio), blood cholesterol, and HbA_1_c levels were obtained. Three recent HbA_1_c levels were averaged for each participant. For those cases diagnosed with blinding DR, HbA_1_c levels at the time of the ocular complication were used, and for no retinopathy controls with DM, HbA_1_c levels immediately before recruitment were averaged. Patients were classified as hypertensive if they were on treatment for hypertension or if they had a blood pressure reading greater than or equal to 140/90 mmHg at the time of recruitment. Hypercholesterolemia was defined as a total cholesterol equal to or greater than 5.5 mmol/l, or current use of lipid lowering medication. Nephropathy was defined as the presence of microalbuminuria (30–300 mg/day) or macroalbuminuria (>300 mg/day). DNA was extracted from peripheral blood samples using the QiaAmp Blood Maxi Kit (Qiagen, Valencia, CA).

Using the tagger program implemented in Haploview 4.0 [25] tag single nucleotide polymorphisms (SNPs) across the CA gene, including the promoter region were selected. SNPs were selected on the basis of linkage disequilibrium patterns observed in the Caucasian (CEU) samples that were genotyped as a part of the International HapMap Project [26]. Only SNPs with minor allele frequency greater than 5% in HapMap were considered. Ten tag SNPs which captured all alleles with an r^2^ of at least 0.8 (mean r^2^=0.963), were genotyped in all individuals using iPLEX Gold chemistry on an autoflex Mass Spectrometer (Sequenom, San Diego, CA).

SNP genotyping was checked for compliance with the Hardy–Weinberg equilibrium using a χ^2^ test. Genotypic associations were assessed in SNPstats [27]. Dominant and recessive models were considered with respect to the minor allele. Haplotypic associations were undertaken in HaploStats (version 1.2.1) [28]. The study was designed to have at least 80% power to detect SNP associations with odds ratios of approximately 1.5, assuming a disease prevalence of 60% among individuals with diabetes, a marker allele frequency of 0.15, and moderate LD between the marker and disease locus.

## Results

Of the 554 participants recruited for this study, 281 participants had no DR and 273 had DR. Of the participants with DR, the grading for the worse affected eye was used in the analyses, with a hierarchy PDR considered worse than NPDR. CSME was also considered separately as an independent analysis. There were 215 participants classified as having blinding DR: 23 participants with severe NPDR, 132 with PDR, and 93 with CSME. Some individuals fell into more than one group of DR as CSME can jointly occur with any of the other DR gradings. If either eye had CSME irrespective of other DR gradings, the patient was classified as having CSME.

Subjects with T1DM and no DR had a significantly lower age, shorter disease duration, lower HbA_1_c, lower rates of nephropathy and hypertension compared to the T1DM cases with blinding DR. Patients with T2DM and no DR were significantly more likely to be female and have shorter disease duration, lower HbA_1_c levels, lower BMI readings and lower rates of nephropathy when compared to participants with T2DM and blinding DR (Table 1).

**Table 1:** Comparison of clinical characteristics of participants by type of diabetes and DR status.

**Clinical characteristics**	**T1DM**		**p value**	**T2DM**		**p value**
	**No DR (n=94)**	**Blinding DR (n=76)**		**No DR (n=187)**	**Blinding DR (n=139)**	
Sex (female)	48 (51)	29 (45)	0.478	100 (53)	45 (35)	0.001
Age (years)	38.4±14.9	49.8±15.5	<0.001	64.3±14.5	64.0±10.7	0.853
Disease duration (years)	15.4±9.1	30.9±13.4	<0.001	12.9±8.7	17.5±8.6	<0.001
HbA_1_c (%)	7.6±2.5	8.8±2.3	0.004	6.6±2.9	7.5±3.4	0.014
BMI (kg/m2)	25.9±7.1	25.9±9.8	0.958	32.2±9.1	29.6±11.5	0.026
Hypercholesterolemia (%)	33 (35)	32 (50)	0.062	120 (64)	81 (63)	0.802
Nephropathy (%)	40 (15)	29 (45)	<0.001	43 (23)	46 (36)	0.014
Smoker (%)	44 (47)	35 (55)	0.331	99 (53)	69 (53)	0.924
Hypertension (%)	37 (39)	46 (72)	<0.001	153 (82)	107 (83)	0.796

Results are presented as number of subjects (%) or mean±standard deviation. P-values for comparison between cases and controls are given.

### SNP analysis

All SNPs were in Hardy–Weinberg Equilibrium in all groups. Genotype counts of individuals with DM and no DR were compared to patients with each type of DR. Genotype frequencies for blinding DR are given in Table 2 and were similar between type 1 and 2 diabetes. No association was found between any *CA* SNP and blinding DR (Table 3), nor the other DR subsets (severe NPDR, PDR, or CSME, data not shown) in combined DM and for T1DM or T2DM analyzed independently. The results remained non-significant in the multivariate analyses after adjusting for disease type, sex, duration of disease or HbA_1_c. A separate haplotype analysis was undertaken for CA polymorphisms and blinding DR (Table 4), NPDR, PDR, or CSME. Statistically insignificant associations were found in the combined DM and for separate T1DM and T2DM analyses.

**Table 2:** Genotype frequencies by type of diabetes and DR status.

	**SNP**	**Genotype**	**T1DM No DR n (%)**	**T1DM Blinding DR n (%)**	**T2DM No DR n (%)**	**T2DM Blinding DR n (%)**
1	rs2403104	TT	48 (53)	36 (57)	78 (43)	60 (47)
		TG	34 (37)	21 (33)	88 (48)	54 (42)
		GG	9 (10)	6 (10)	16 (9)	15 (12)
2	rs17741410	AA	60 (65)	42 (67)	131 (72)	91 (70)
		AG	32 (34)	18 (29)	46 (25)	35 (27)
		GG	1 (1)	3 (5)	5 (3)	4 (3)
3	rs1496533	TT	22 (24)	12 (19)	44 (24)	34 (26)
		TC	47 (51)	32 (52)	93 (51)	62 (48)
		CC	24 (26)	18 (29)	44 (24)	34 (26)
4	rs17814594	TT	58 (65)	45 (73)	131 (72)	89 (70)
		TC	30 (34)	15 (24)	46 (25)	36 (28)
		CC	1 (1)	2 (3)	4 (2)	3 (2)
5	rs12544332	AA	29 (31)	23 (37)	60 (33)	43 (33)
		AC	45 (48)	30 (48)	89 (49)	60 (47)
		CC	19 (20)	9 (15)	33 (18)	26 (20)
6	rs1496529	AA	56 (60)	41 (65)	98 (53)	69 (53)
		AG	30 (32)	18 (29)	77 (42)	52 (40)
		GG	8 (9)	4 (6)	9 (5)	9 (7)
7	rs725605	TT	24 (26)	20 (31)	49 (27)	41 (32)
		TC	47 (50)	31 (48)	93 (51)	62 (48)
		CC	23 (24)	13 (20)	40 (22)	27 (21)
8	rs2645050	AA	60 (65)	44 (71)	131 (72)	90 (70)
		AG	32 (34)	16 (26)	45 (25)	36 (28)
		GG	1 (1)	2 (3)	5 (3)	3 (2)
9	rs2645049	CC	54 (58)	37 (59)	93 (51)	70 (54)
		CT	27 (29)	19 (30)	80 (44)	51 (40)
		TT	12 (13)	7 (11)	9 (5)	8 (6)
10	rs13278559	CC	78 (84)	54 (86)	147 (81)	105 (81)
		CT	15 (16)	8 (13)	33 (18)	24 (18)
		TT	0 (0)	1 (2)	2 (1)	1 (1)

Results are presented as n (%).

**Table 3:** P-values for association of CA tag SNPs with blinding DR by type of diabetes under dominant and recessive genetic models.

**SNP**	**Combined DM**		**T1DM**		**T2DM**	
	**Dominant**	**Recessive**	**Dominant**	**Recessive**	**Dominant**	**Recessive**
rs2403104	0.46	0.31	0.73	0.92	0.54	0.36
rs17741410	0.74	0.79	0.99	0.54	0.70	0.89
rs1496533	0.77	0.75	0.68	0.93	0.85	0.85
rs17814594	0.83	0.91	0.74	0.80	0.65	0.99
rs12544332	0.87	0.73	0.48	0.44	0.82	0.94
rs1496529	0.82	0.99	0.36	0.60	0.74	0.80
rs725605	0.23	0.68	0.51	0.33	0.26	0.98
rs2645050	0.93	0.98	0.95	0.76	0.92	0.85
rs2645049	0.83	0.78	0.87	0.45	0.93	0.84
rs13278559	0.84	0.45	0.51	0.23	0.55	0.89

Results have been adjusted for sex, diabetes type, duration of disease, and HbA_1_c.

**Table 4:** Frequency of common haplotypes and association with blinding DR under additive and dominant genetic models.

**T1DM**															
**Haplotype**	**1**	**2**	**3**	**4**	**5**	**6**	**7**	**8**	**9**	**10**	**Haplotype frequency**	**No DR frequency**	**Blinding DR frequency**	**Additive p value**	**Dominant p value**
1	T	G	T	C	C	A	C	G	C	C	0.165	0.175	0.151	0.404	0.333
2	G	A	T	T	C	G	C	A	T	C	0.205	0.218	0.184	0.429	0.616
3	T	A	C	T	A	A	T	A	C	C	0.428	0.410	0.459	0.203	0.228
**T2DM**															
**Haplotype**	**1**	**2**	**3**	**4**	**5**	**6**	**7**	**8**	**9**	**10**	**Haplotype frequency**	**No DR frequency**	**Blinding DR frequency**	**Additive p value**	**Dominant p value**
1	G	A	T	T	A	A	T	A	C	T	0.041	0.045	0.034	0.822	0.818
2	T	A	C	T	A	A	T	A	C	T	0.050	0.046	0.051	0.504	0.502
3	T	G	T	C	C	A	C	G	C	C	0.151	0.143	0.162	0.556	0.466
4	G	A	T	T	C	G	C	A	T	C	0.224	0.220	0.229	0.516	0.540
5	T	A	C	T	A	A	T	A	C	C	0.397	0.392	0.410	0.863	0.941

SNPs are numbered as in Table 2 and the p-value for all haplotypes with a frequency >2% are given. Results have been adjusted for sex, diabetes type, duration of disease and HbA_1_c.

## Discussion

The pathogenesis of DR is complex, with few independent risk factors identified other than duration and extent of hyperglycemia and systemic hypertension. Increased carbonic anhydrase levels have been described in the vitreous of patients with PDR [23]. Functional evidence from rat models supports a potential role for CA in the pathogenesis of DR [23]. It is postulated that CA is released from lysed blood cells as a result of retinal and vitreous hemorrhage. Increase of vitreous pH as a result of CA activity is believed to increase vascular permeability through activation of the kallikrein-kinin system [23]. This increase in vascular permeability is likely to result in edema and subsequent damage to the retina, contributing to DR (and especially macular edema) and vision loss. A pilot study of macular edema treatment with acetazolamide showed significant improvement in fluorescein-angiographic findings and perimetric data, further supporting the role of carbonic anhydrase in DR development [29]. In these functional studies, proteins have been assayed at a late time point in the disease process, as vitreous was removed from patients during vitrectomy as a treatment for advanced disease. In addition, the role of CA in these studies has been associated with sight threatening DR. It is therefore possible that CA is involved only in end stage damage to the retina, such as that of PDR and CSME and not in NPDR. Thus it is difficult to know if the CA in vitreous increases as a cause or consequence of DR.

To our knowledge, this is the first report investigating *CA* sequence variation as a risk factor for DR. Our results suggest that common sequence variation in *CA* is not a major risk factor for the development of DR in either T1DM or T2DM. It is acknowledged that participants with DR had the presence of increased risk factors (including older age, longer diabetes duration, higher HbA_1_c levels, and higher rates of nephropathy) when compared to those without DR. However an attempt to control for their effects on DR development was made in the multivariate analyses. While this study was powered to detect a modest effect size odds ratio (OR=1.5), the study design does not allow us to exclude a rare *CA* polymorphism, or a series of separate uncommon pathogenic mutations as a contributor to DR development in a small subset of DR subjects. It is also possible that other factors, perhaps different genetic loci, may play a role in the regulation of *CA*. It remains to be determined by clinical studies whether *CA* represents a valid target for the treatment or prevention of DR.

Further research is required to obtain a better understanding of DR pathogenesis and to decrease this global burden of disease.
